# Predicting Hospice Transitions in Dementia Caregiving Dyads: An Exploratory Machine Learning Approach

**DOI:** 10.1093/geroni/igac051

**Published:** 2022-08-11

**Authors:** Suzanne S Sullivan, Wei Bo, Chin-Shang Li, Wenyao Xu, Yu-Ping Chang

**Affiliations:** School of Nursing, University at Buffalo, Buffalo, New York, USA; Department of Computer Science Engineering, University at Buffalo, Buffalo, New York, USA; School of Nursing, University at Buffalo, Buffalo, New York, USA; Department of Computer Science Engineering, University at Buffalo, Buffalo, New York, USA; School of Nursing, University at Buffalo, Buffalo, New York, USA

**Keywords:** Dementia, NHATS, Precision health, Serious illness planning, Social determinants of health

## Abstract

**Background and Objectives:**

Hospice programs assist people with serious illness and their caregivers with aging in place, avoiding unnecessary hospitalizations, and remaining at home through the end-of-life. While evidence is emerging of the myriad of factors influencing end-of-life care transitions among persons living with dementia, current research is primarily cross- sectional and does not account for the effect that changes over time have on hospice care uptake, access, and equity within dyads.

**Research Design and Methods:**

Secondary data analysis linking the National Health and Aging Trends Study to the National Study of Caregiving investigating important social determinants of health and quality-of-life factors of persons living with dementia and their primary caregivers (*n* = 117) on hospice utilization over 3 years (2015–2018). We employ cutting-edge machine learning approaches (correlation matrix analysis, principal component analysis, random forest [RF], and information gain ratio [IGR]).

**Results:**

IGR indicators of hospice use include persons living with dementia having diabetes, a regular physician, a good memory rating, not relying on food stamps, not having chewing or swallowing problems, and whether health prevents them from enjoying life (accuracy = 0.685; sensitivity = 0.824; specificity = 0.537; area under the curve (AUC) = 0.743). RF indicates primary caregivers’ age, and the person living with dementia’s income, census division, number of days help provided by caregiver per month, and whether health prevents them from enjoying life predicts hospice use (accuracy = 0.624; sensitivity = 0.713; specificity = 0.557; AUC = 0.703).

**Discussion and Implications:**

Our exploratory models create a starting point for the future development of precision health approaches that may be integrated into learning health systems that prompt providers with actionable information about who may benefit from discussions around serious illness goals-for-care. Future work is necessary to investigate those not considered in this study—that is, persons living with dementia who do not use hospice care so additional insights can be gathered around barriers to care.

**Translational Significance:** This article reveals an array of critical quality-of-life and social determinant of health factors that may influence the use of hospice services in dementia caregiving dyads over time. This exploratory research using machine learning approaches lays the foundation for future research and the development of personalized approaches for addressing the end-of-life care needs and assisting with transitions to hospice care of persons living with dementia and their caregivers. Data driven models, when translated into practice, may help achieve the vision of precision health and learning health systems by reducing structural barriers to hospice care transitions and promoting end-of-life health equity.

## Background and Objectives

Alzheimer’s disease and related dementias are a group of progressive, life-limiting cognitive and physical conditions that affect an estimated 55 million people and their informal family caregivers worldwide (World Health Organization [WHO], 2021). Although dementia is widely prevalent, particularly among older adults, those with social disparities are disproportionately affected by the condition (Alzheimer’s Association [AA], 2021]). The physical, financial, and emotional burdens of dementia are profound and affect the quality-of-life of persons living with dementia and their families, particularly as cognitive decline accelerates and death approaches (Vick et al., 2019). Yet persons living with dementia and their caregivers are unlikely to receive external support despite the sometimes-overwhelming distress experienced during end-of-life dementia caregiving (Luth et al., 2021; Ornstein et al., 2017; Vick et al., 2019). Thus, the treatment and care of persons living with dementia have broad and deleterious affects on society as a whole, as caregivers of persons living with dementia in the last year of life are typically unpaid spouses who are caregiving alone (Ornstein et al., 2019).

Hospice programs assist people with serious illness and their caregivers with aging in place, avoiding unnecessary hospitalizations, and remaining at home through the end-of-life process (Luckett et al., 2013). Indeed, nearly half of all hospice patients have a primary or comorbid diagnosis of dementia (Aldridge et al., 2021). Increased use of hospice care, therefore, could help reduce the burdens of end-of-life caregiving for some families, and potentially prevent nonbeneficial hospitalizations and emergency department use among persons living with dementia in the years and months leading up to death (Wright et al., 2018). Although hospice use is increasing nationwide (Teno et al., 2018), this essential care remains underutilized among many persons living with dementia who are qualified to receive this support. It is unclear why this is the case; however, it is known that disparities persist, particularly among vulnerable populations.

Research reveals that social, economic, political, and structural barriers may perpetuate end-of-life health disparities (Hart et al., 2018; Starr et al., 2021). For instance, having Medicaid reduces the likelihood of hospice referrals, while physician practice patterns are one of the strongest predictors of hospice use (Obermeyer et al., 2015; Starr et al., 2021). Deploying systematic approaches at the level identifying persons living with dementia who may benefit from hospice care may help overcome some of these barriers by influencing practice patterns that encourage discussions pertaining to end-of-life caregiving needs. However, the vision of precision health within learning health systems, where care decisions are informed and guided by knowledge of personalized risk profiles, is nascent, particularly within the domain of end-of-life caregiving, but they are promising (Cardona et al., 2022; Gambhir et al., 2018). For example, researchers have used machine learning approaches to develop a recommendation system of nursing home residents interests and preferences to enhance person-centered care (Gannod et al., 2019). During the coronavirus disease 2019 (COVID-19) pandemic, researchers developed a highly accurate mortality risk prediction model that enabled the prioritization of care for those at the most risk for experiencing poor health outcomes (Gao et al., 2020). Recently, researchers have developed, and pilot tested an online conversation guide that is compatible with electronic health record software that enables health care providers to screen persons living with dementia for short-term mortality risk and elicit their values and preferences around treatment options (Cardona et al., 2022). However, this work is in the early stages, so it is necessary to further the science by developing additional personalized models that can guide clinical practice.

Quality-of-life is a personal, subjective experience, so using quality-of-life features of family caregivers and persons living with dementia nearing the end-of-life may help to better identify those who could benefit from additional support. However, quality-of-life, particularly near the end-of-life, is often influenced by social context (Corpora, 2021). The World Health Organization (WHO) defines the social determinants of health (SDH) as “non-medical factors that influence health outcomes” that are influenced by forces such as social norms, economic, and political policies, and systems that shape the conditions of daily life (WHO, 2022). In other words, quality-of-life is directly influenced by SDH. Nevertheless, the intersection of quality-of-life and SDH is a surprisingly understudied area among persons living with dementia, despite the known burdens, stigma, and social affects of end-of-life dementia caregiving (Starr et al., 2021).

Quality-of-life as a predictor of hospice use is also understudied even though it is known that hospice care can improve quality-of-life of persons living with dementia and their caregivers (Institute of Medicine [IOM], 2015). For example, it is known that substantial declines in quality-of-life and well-being can be identified up to 5 years prior to death (Cohen-Mansfield et al., 2018). However, research tying quality-of-life to end-of-life care is currently focused on physical conditions, such as the presence of chronic illness and symptom burdens, rather than more broadly on social and other factors potentially influencing quality-of-life (Cagle et al., 2020; Feder et al., 2020). Moreover, poor quality-of-life is associated with goals-of-care discussions, having a high quality-of-life may also inhibit the likelihood of advance directive completion (Tang et al., 2018). Because persons living with dementia and their caregivers often have similar social needs, they likely also influence the quality-of-life of one another bidirectionally. Therefore, dyadic models describing the association between quality-of-life within the context of SDH and hospice care may help further knowledge, policy, and practice to improve care for persons living with dementia nearing the end-of-life (Axelsson et al., 2020).

This study aims to identify critical quality-of-life and SDH factors in a population-level data set of persons living with dementia and their primary caregivers in dyads that are associated with hospice use via secondary data analysis of the National Health and Aging Trends Study (NHATS) linked to the National Study of Caregiving (NSOC). This study employs cutting edge research approaches (machine learning) because these inductive, hypothesis-generating methods are specifically designed to identify complex nonlinear patterns in data that can reveal novel insights. Machine learning generates knowledge and new “ways of knowing” and can pave the way for future research and the eventual development of predictive algorithms that learn from new data (Corwin et al., 2019). Significantly, once fully developed, precision health models within learning health systems have the capacity to guide practice by alerting clinicians of potential care needs for persons living with dementia and their family caregivers.

## Research Design and Methods

This multiyear secondary data analysis of the NHATS linked to the NSOC received human subject approval from our university Institutional Review Board (IRB) #MODCR00005076. The data are used with the permission of the NHATS principal investigators under a written data use agreement.

### Data Sets

The NHATS and NSOC studies are prospective, longitudinal, annual surveys conducted among Medicare beneficiaries aged 65 years and older and their caregivers across the United States (National Health and Aging Trends Study [NHATS], n.d.). The NHATS contains data around key concerns for 12,427 (2011–2019) older adults and 3,305 primary caregivers. Both studies are sponsored by the National Institutes of Aging (NIA; NIA U01AG32947) and are conducted at The Johns Hopkins University. Our method for linking the NHATS/NSOC longitudinally and identifying persons living with dementia within the linked files is published elsewhere (Sullivan et al., 2022).

The NHATS covers an array of important domains for determining health and disability trends concerning older adults in the United States (NHATS, n.d.). Content areas include questions related to physical condition, social needs, cognitive capacity (including dementia), self-care activities and capacity, medical care, participation in valued activities, and well-being. Other content areas include the presence of chronic conditions, symptoms, sensory impairments, subjective and economic well-being, and demographics. The last month of life interview focuses on the quality of end-of-life care.

The NSOC is a companion study to the NHATS of up to five family and unpaid caregivers of a subset of study participants (NHATS, n.d.). Caregivers are interviewed on activities, the duration and intensity of care, effects of caregiving on social participation and well-being, and demographic factors. The NSOC corresponds with the NHATS timeline and caregiver interviews were conducted in 2011, 2015, and 2017.

### Theoretical Frameworks

This study is guided by a socialecological framework, which accounts for the interactions between individuals and their environment that affect health (Bronfenbrenner, 1979). Socialecological models are consistent with the WHO definition of SDH (WHO, 2022).

Feature selection was guided from the perspective that quality-of-life within the social context may be an important, yet understudied, predictor of hospice utilization. Commonly used quality-of-life frameworks have long included SDH items such as social, environmental, financial, and health care access needs. We employ quality-of-life frameworks, in efforts to provide a new lens through which to investigate associations with hospice care use.

The McGill End-of-Life Quality-of-Life (MQOL-E) and related Quality-of-Life in Life Threatening Illness-Family Caregiver Version (QOLLTI-F) for caregivers framed feature selection (Axelsson et al., 2020; Cohen et al., 2017). The MQOL-E domains include overall quality-of-life, physical, psychological, existential, social, burden, environment, cognition, and access to health care domains. Overall quality-of-life, environment, patient condition, caregivers’ own state, outlook, quality-of-care, relationships, and financial worries are domains within the QOLLTI-F.

Specific to this study, items related to quality-of-life and SDH included caregiver physical condition, cognitive status, emotional and social needs and persons living with dementia physical and cognitive condition, psychological and cognitive features, social situation, medical care, relationships, and existential needs. Sociodemographic items (e.g., education, socioeconomic status, and geographic location) of both the persons living with dementia and their caregiver were considered (Table 1).

**Table 1.** Candidate Predictive Variables by Features Related to Quality-of-Life and Social Determinants of Health (SDH) of Persons Living With Dementia and Their Primary Caregiver

Variable	NHATS item	NSOC item	Variable description
Persons living with dementia candidate variables			
Physical conditions and symptoms	hc#hartsrgyr		Heart surgery past 12-months
	hc#disescn7		Has lung disease
	hc#disescn2		Has heart disease
	hc#disescn6		Has diabetes
	hc#disescn8		Had stroke
	hc#disescn10		Has cancer
	hc#sleepmed		Sleep medication use
	ss#probspeak		Problems speaking
	ss#probchswl		Problems chewing or swallowing last month
	sc#eathlp		Needing help to eat
	hc#health		Overall health
	ss#painlimts		Pain ever limits activity
	ss#prbbrlimt		Breathing problems limit activity
	cca#hwofthom		How often help getting around home
	hc#aslep30mn		Over 30 min to fall asleep
	hw#lst10pnds		Lost 10 pounds in last year
Psychological/cognitive features	hc#depresan1		Little interest or pleasure
	hc#depresan2		Down, depressed, or hopeless
	hc#depresan3		Feeling nervous, anxious
	hc#depresan4		Unable to stop worrying
	cg#ratememry		Rate your memory
	mc#medsmis		Make mistake taking meds
Existential needs	pa#hlkpgoenj		Health prevents enjoying life
	wb#offelche3		Feeling full of life
	wb#truestme3		Given up improving life
	wb#agrwstmt1		Others to determine activities
	hc#worrylimt		Worry ever limit activities
Social situation	mo#outoft		How often go outside
	fl#noonetalk		No one to talk to
	sn#dnumsn		Number in social network
	pa#htkfrrlsr		Health kept from religious services
	pa#hlkepfvst		Health kept from visiting family friends
	pa#hlkpfrvol		Health kept from volunteering
Caregiver candidate variables			
Caregiver features (physical, social, and emotional)		che#enrgylmt	Energy often limited
		cac#diffphy	Caregiver physical difficulty helping
		cac#exhaustd	Caregiver exhausted at night
		cac#toomuch	Care more than can handle
		cac#uroutchg	Care routine then changes
		cac#notime	No time for self
		cac#diffemlv	Caregiver emotional difficulty
		cpp#hlpkptgo	Kept from going out
		che#health	General health
		che#sleepint	Interrupted sleep
		op#numhrsday	Number of hours per day help
		op#numdaysmn	Number of days help per month
Sociodemographic features (persons living with dementia and caregiver)		op#leveledu	Caregiver education
		op#age	Caregiver age
		cac#diffinc	Caregiver financial difficulties
		op#relatnshp	Caregiver relationship
		r#d2intvrage	Persons living with dementia age
		r#dgender	Persons living with dementia gender
		SPrace	Persons living with dementia race: 1 = White, 2 = African American, 3 = other
		hh#martlstat	Persons living with dementia marital status
		re#dcensdiv	Persons living with dementia census division
		hh#dhshldchd	Persons living with dementia number of children
		ia#totinc	Persons living with dementia total income
		ew#progneed1	Persons living with dementia received food stamps
		ew#finhlpfam	Persons living with dementia financial help from family
		mc#havregdoc	Persons living with dementia have a regular doctor
		hc#hosptstay	Persons living with dementia hospital stay in last 12-months
		hc#hosovrnht	Persons living with dementia number of hospital stays

*Notes*: Persons living with dementia and their primary caregiver from the National Health and Aging Trends Study (NHATS) and National Study of Caregiving (NSOC); # indicates NHATS/NSOC round number.

### Population

NHATS participants with dementia (either possible or probable) were included. Caregivers who provided the greatest number of hours of care to the persons living with dementia within the month prior to the NSOC interview were determined to be the primary caregiver.

### Approach

A multiyear, secondary analysis of NHATS/NSOC linked caregiver/care recipient dyads to determine if there is a relationship between quality-of-life, SDH, and hospice use was conducted using machine learning to identify important features on a linked data set of NHATS rounds 5, 7, and 8 (for death) and NSOC rounds 5 (2015) and 7 (2017), with the baseline year (2015).

This exploratory study utilizes multiple machine learning approaches (i.e., correlation matrix analysis, principal component analysis [PCA], information gain ratio [IGR], and random forest [RF]) to reveal data features that are most important for predicting hospice utilization. This multipronged approach is necessary because there is no unified machine learning method for use with the differing types of data that make up the features in the present study. Because machine learning methods are inductive, and help to generate hypotheses, this multistep approach makes it possible to exclude variables that all or most methods deem unimportant, which helps to narrow the feature space. Agreement between models on important features also helps determine the starting point for constructing future models rather than working with the raw data. Please see Supplementary Figure 1 outlining the steps for screening out the data of interest.

#### Hospice

The target variable (pd8hospcelml) indicated hospice use in the last month of life interview within the NHATS from round 8 (2018). Hospice was dichotomized as 1 = yes and 0 = no from its’ original coding provided by NHATS (1 = yes and 2 = no).

#### Population

NHATS participants with either probable or possible dementia and their primary caregiver were selected based on the dementia classification in round 7 data. Note that it was possible for some NHATS participants not to have dementia in round 5. Next, persons living with dementia who died in round 8 were identified because the hospice feature is only applicable to decedents in the NHATS data set. All persons living with dementia who did not use hospice in round 8 were deleted. The final data set included 117 persons living with dementia, 65 of them received hospice care, and 52 of them did not.

#### Candidate predictor variables

Candidate variables were identified using two interrelated quality-of-life frameworks for caregivers (QOLLTI-F) and persons living with dementia (MQOL-E). Sixty one candidate features were collected under the following domains: physical condition and symptoms, psychological/cognitive features, existential needs, social situation, caregiver features (physical, emotional, and social), and sociodemographics of the person living with dementia and their primary caregiver (Table 1). The original coding provided by NHATS/NSOC for dichotomous predictor variables was retained (yes = 1 and no = 2).

#### Analytical and predictive framework

Multiple approaches were taken to explore whether there was a difference in the relationship between the predictive variables in round 5 or round 7 data and the target variables (Supplementary Figure 1 and Supplementary Table 1). Where differences existed, features were selected for multiyear analysis to reveal more insights. First, independence analysis was conducted using Pearson’s correlation heatmap matrices to reduce redundancies and reveal correlation insights among variables (Supplementary Figure 2a and b). Then data were visualized using PCA (Supplementary Figure 3). Next, features were selected using IGR and RF. Finally, prediction models were constructed by using the RF algorithm, and several results were compared.

##### Variable independence analysis

Pearson’s correlation matrices were displayed as heatmaps for both rounds (5 and 7) to visualize the data and help determine variable independence (Supplementary Figure 2a and b). The Pearson’s correlation coefficient measures the linear relationship between two continuous random variables. The output ranges from −1 to +1, with 0 representing no linear correlation, negative values being negative correlation, and positive values being a positive correlation. Correlation coefficients with *p* < .05 were considered significant. Content experts (first and last author) visualized the heatmaps and identified variables indicating strong correlations (absolute Pearson’s correlation coefficient 0.6–1.0) and selected the question best representing the concept of interest to reduce conceptual redundancies among highly correlated items. The negative values in the data set were not dealt with in this step.

##### Data set visualization

The two rounds of data were analyzed together by PCA to better understand and reveal the deeper relationships between variables (Supplementary Figures 3 and 4). PCA was conducted on rounds 5 and 7 together to find the most important aspects of the predictive variables. Although PCA is mainly used for dimensionality reduction, it is reasonable here to help visualize and analyze which predictive variables are more important by contributing the most to the first and second principal components in our study. PCA is conducted in three steps: (a) scree plot represents the contribution rate of each principal component, (b) quality of representation (higher squared cosine) which indicates variable importance, and (c) contribution of variables (larger contributions to variability which suggest a greater contribution to the principal component).

The scree plot represents the contribution rate of each principal component, followed by how many principal components to choose (Supplementary Figure 3). The squared cosine represents the quality of representation for variables on the factor map, and its value is to measure the usefulness of a variable (Supplementary Figure 3). The higher the value, the more important the variable is in the PCA. A high squared cosine value indicates a good representation of the variable on the principal component, and the variable is positioned close to the circumference of the correlation circle. The contribution of a variable in calculating the variability for a given principal component is expressed as a percentage (Supplementary Figure 4). The variables associated with the first and second principal components usually are the most important in explaining the variability of the data set. The larger the contribution value, the greater the contribution of the variable to the principal component.

##### Feature selection

The original data set contains many negative and N/A values, and the sample is small, so it was necessary to preprocess the data prior to feature selection. To address the small sample size, negative values were converted into N/A values, and the N/A values were processed according to the type of feature. For continuous features, N/As were replaced with column medians. The most frequent levels replaced categorical feature N/As.

Two methods were conducted in the feature selection step: IGR and RF. Because most predictive variables were categorical, IGR analyses were conducted based on round 5 and round 7 data individually, and both rounds of data together (Supplementary Figure 4). Information gain is an effective measure of how much information a categorical feature provides about a class (the feature with a larger number means a larger effect on classification). IGR was first applied to round 5 and round 7 data, respectively. Also, for the aim of multiyear analysis, additional IGR was applied to round 5 and round 7 data together.

Next, we used the RF algorithm to calculate feature importance, then selected several important features based on the results. RF is an algorithm that integrates multiple trees through the idea of ensemble learning, its basic unit is a decision tree. RF uses random resampling and random node splitting techniques to generate multiple decision trees and relies on the voting selection of decision trees to determine the final classification result. In our study, the Gini coefficients were used as a criterion to train the RF models. Considering the smaller sample size, fivefold cross-validation is applied to obtain average and robust results. Then each feature was sorted from largest to smallest based on its importance value. Due to the large number of features, only the top 10 features are considered. Fivefold cross-validation provided the 50 most important features (including duplicates). Through frequency statistics, features with a frequency equal to or more than three were screened out as important features.

#### Prediction and analysis

Four different RF prediction models with fivefold cross-validation were constructed.

##### Baseline prediction models

To better compare the results and reveal the necessity of multiyear analysis, three baseline prediction models were built on round 5 data, round 7 data, and two rounds’ data together. For the two rounds’ data model, features from round 5 and round 7 were regarded as different.

##### Prediction model based on RF important features

In the feature selection step, by using RF to calculate the importance of features, 10 features were screened out through the frequency criterion. A new RF model was built using only these 10 important features.

##### Prediction model based on IGR important features

In the feature selection step by using IGR to calculate the importance of features, seven features were screened out through the frequency criterion. Another new RF model was built using only these seven important features.

##### Correlation analysis for interpreting final results

In the final step, a correlation analysis was conducted using Kendall’s rank correlation coefficients to interpret the final results of the important features selected by IGR. Kendall’s rank correlation coefficient is a nonparametric (distribution-free) rank statistical parameter, which is used to measure the strength of the monotonic relationship between two ordered variables, among categorical variables.

#### Chi-squared tests for association between Medicaid, residence, and hospice use

In a separate analysis, we sought to further explore the association between Medicaid, persons living with dementia residence (community vs. residential care facility), and hospice use using Pearson’s chi-squared tests. Residential care settings at the time of death in NHATS include but are not limited to nursing homes. The entire NHATS sample comprises Medicare recipients, so we assume that 100% of the sample has Medicare.

## Results

This study comprised NHATS rounds 5, 7, and 8 (for death), and NSOC rounds 5 and 7. There were 61 candidate quality-of-life and SDH predictors of hospice among 117 persons living with dementia and their primary caregivers. Sixty percent of persons living with dementia were female (*n* = 70). The majority were identified as race “other” 80 (68%), 28 (24%) persons living with dementia were White, and 9 (8%) were African Americans. The “other” category includes persons with more than one race or if race was indeterminant. The person living with dementia’s age ranged from 65 to 89 years and older in rounds 5 and 7. Primary caregivers were aged 47.5 (range 27–89+) years old, on average, in round 5, and 52 (range 25–89+) years old in round 7.

Independence analysis and data set visualization revealed that the features of rounds 5 and 7 have different effects on hospice use, which prompted further analysis. Two different feature selection methods (IGR and RF) obtained 7 and 10 important features, respectively. Two RF prediction models were established for these important features and compared with the three baseline models. Robust prediction results obtained indicate multiyear analysis is necessary and can effectively identify important features associated with hospice use.

### Exploratory Data Analysis and Feature Selection

#### Variable independence analysis

##### Pearson’s correlation

Heatmaps of the 61 features and the target variable (hospice) in rounds 5 and 7 with coefficients whose *p*-value < .05 are included (Supplementary Figure 2a and b). In round 5, age was positively correlated with hospice (i.e., older persons were more likely to use hospice), and diabetes was negatively correlated with hospice (i.e., people with diabetes are more likely to use hospice). In round 7, eight features were negatively correlated with hospice, and the person living with dementia’s age was positively correlated with hospice. Older persons living with dementia who reported they worry less, feel full of life, that pain limits their activity, health prevents them from enjoying life, health keeps them from attending religious services, and that other people determine most of what they can and cannot do, persons living with dementia who have fewer people in their social network, and those who have a good memory are more likely to receive hospice care. (Note the variable coding hospice [no = 0 and yes = 1] and diabetes [yes = 1 and 2 = no] affects the interpretation, i.e., if diabetes decreases from 2 to 1 [from no to yes], then hospice will increase from 0 to 1 [no to yes]).

#### Data set visualization

##### PCA

As Supplementary Figure 3 shows, only 7.5% of the information (difference) contained in the data is retained by the first principal component. The second principal component retains 6.5% of the information, indicating low redundancy of the raw data set. Although the percentage of explained variance contained in the first two principal components is not high, they are much higher than the other remaining dimensions. This determines whether further analysis of the first two principal components is worthwhile. The squared cosine figure (Supplementary Figure 3) shows features that have larger contribution values on the first principal component are mostly from round 7 and are related to caregivers. Also, their positions are close together, indicating there are correlations among them. For the second principal component, the features from both rounds 5 and 7 have contributions, but they are mostly related to persons living with dementia. Supplementary Figure 4 shows the contributions of the top 10 features in the first and second principal components, respectively, revealing the same results of the squared cosine values.

#### Feature selection

##### IGR

Seven important features, where three are from round 5 (Supplementary Figure 5a) and the other four are from round 7, were identified (Supplementary Figure 5b). Features whose importance was >0.05 were selected due to increased significance in comparison to others. All three figures show receiving food stamps, having trouble chewing or swallowing, and having a regular physician are the most important predictors in round 5 (Supplementary Figure 5a and c). Features indicating persons living with dementia memory, receiving food stamps, whether health prevents enjoying life, and having diabetes are the most important round 7 features (Supplementary Figure 5b and c). Distinguishing features selected by IGR indicate that persons living with dementia memory rating, receiving food stamps, whether health prevents enjoying life, having trouble chewing or swallowing, diabetes, and a regular physician can predict hospice use well (accuracy = 0.685; sensitivity = 0.824; specificity = 0.537; AUC = 0.743; Supplementary Table 2).

##### RF

The top 10 important features selected by fivefold cross-validation in each fold by using both rounds’ data are presented in Supplementary Figure 6. It reflects the frequency of each important feature within the fivefolds. Features with a frequency ≥3 were selected, and 10 important features were obtained from both rounds. The results indicate persons living with dementia, and their primary caregivers’ age, income, and census division are important in both rounds, number of days help per month, and whether health prevents enjoying life are all important in round 7. Different important features were obtained from these two methods because only categorical features were included in the IGR method, in addition to the use of RF and Gini coefficients as criterion. Distinguishing features selected by RF indicate that persons living with dementia and their primary caregivers’ age, income, census division, number of days help per month, and whether persons living with dementia health prevents enjoying life also can predict hospice use well (accuracy = 0.624; sensitivity = 0.713; specificity = 0.557; AUC = 0.703; Supplementary Table 2).

##### Kendall’s correlation coefficients

**Figure 1.**
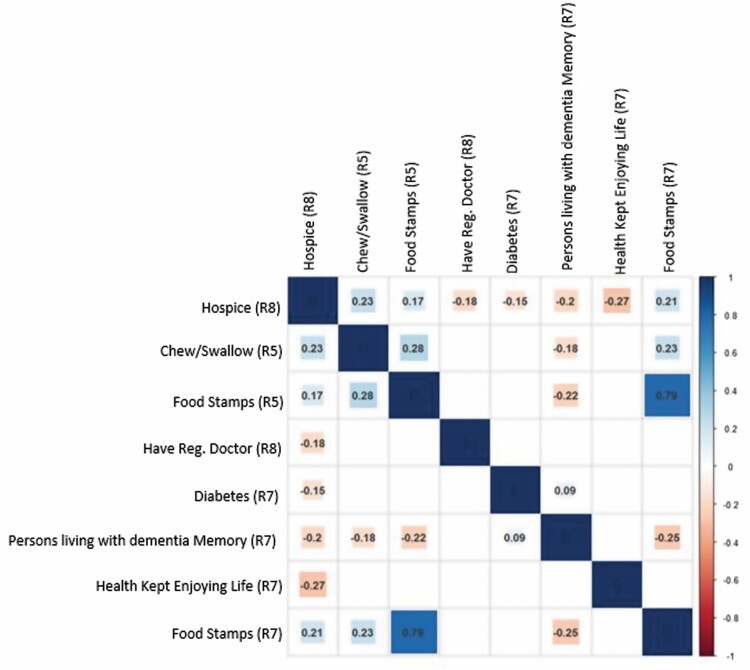
Kendall’s correlation heatmaps for the final information gain ratio (IGR) model.*Notes*: Rounds 5 and 7: Persons living with dementia who have diabetes and a regular physician, who do not receive food stamps, do not have trouble chewing or swallowing, have a good memory, and who report that their health prevents them from enjoying life are more likely to use hospice.

Seven important variables are selected from round 5 and round 7 by IGR. A Kendall’s rank correlation coefficient was calculated to reveal the direction of the relationships between these variables and hospice use. Figure 1 shows the final Kendall’s heatmap with the numbers in the figure indicating the rank correlation coefficient values after removing correlation coefficients with *p*-values > .1.

The variables indicating whether persons living with dementia receive food stamps (rounds 5 and 7) or have trouble chewing or swallowing are positively correlated with hospice, indicating that persons living with dementia who do not receive food stamps, without trouble chewing or swallowing, are more likely to use hospice. Persons living with dementia memory, whether health prevents enjoying life, having a regular physician, and having diabetes are negatively correlated with hospice. This indicates that persons living with dementia who have a good memory, diabetes, a regular physician, and who report that health prevents enjoying life are more likely to utilize hospice services.

#### Chi-squared tests for association between Medicaid, residence, and hospice use

##### Medicaid and residence

There was no significant association between the person living with dementia’s residence prior to death (community versus residential care setting) and whether they had Medicaid insurance (χ ^2^[1] = 0.588, *p* = .443). There were 44 persons living with dementia living in residential care settings, 16 (36.4%) had Medicaid and 28 (63.6%) did not. Among persons living with dementia in the community (*n* = 62), 17 (27.4%) had Medicaid and 45 (72.6%) did not.

##### Medicaid and hospice

There was a significant association between hospice use and Medicaid (χ ^2^[1] = 4.225, *p* = .040). Among 59 persons living with dementia who accessed hospice care, 13 (22%) had Medicaid and 46 (78%) did not.

##### Hospice and residence

There was no significant association between whether the persons living with dementia used hospice or not and their residence (community versus residential care setting) prior to death (χ ^2^[1] = 0.011, *p* = .917). Among 49 persons living with dementia who lived in residential care settings, 28 (57.1%) accessed hospice care and 21 (42.9%) did not. In the community, 37 (54.4%) persons living with dementia accessed hospice and 31 (45.6%) did not.

##### Medicaid, residence, and hospice

Among persons living with dementia who lived in the community at the time of death (*n* = 62), there was no significant association between hospice use and Medicaid status (χ ^2^[1] = 1.871, *p* = .171). There was also no significant association found between hospice use and Medicaid status among persons living with dementia who lived in a residential care setting at the time of death (*n* = 44; χ ^2^[1] = 1.367, *p* = .242).

## Discussion and Implications

This study uses machine learning approaches identifying quality-of-life and SDH features within a socioecological framework that predicts hospice use among persons living with dementia and their primary caregivers, which can be detected up to three years prior to death. Two final models of distinguishing features were identified.

### IGR Final Model

The IGR final model reveals six important features associated with hospice use: diabetes, chewing or swallowing problems, food stamps, having a regular physician, health preventing enjoying life, and memory rating of the person living with dementia.

Having a physical condition such as diabetes predicts hospice use but chewing, and swallowing problems were not associated with hospice. This is consistent with prior research linking dementia onset and survival time to diabetes which reflects that the onset of diabetes and death occurs more than 2 years earlier in persons living with dementia, on average, compared to those without diabetes; particularly, when dementia is diagnosed before age 65 or when an individual has had dementia for above 15 years (Zilkens et al., 2013). Research also suggests the incidence of severe hypoglycemia and physical frailty is associated with dementia and mortality risk (Han et al., 2022). Effective diabetic management (blood glucose monitoring, adherence to treatment plans, and medication management) is dependent on cognitive abilities, and poorly managed diabetes leads to microvascular complications; thus, persons living with dementia have an increased risk of poor health outcomes and decline over those without the condition (Santos et al., 2018).

Swallowing difficulties contribute to approximately one-quarter of all deaths among those with Lewy Body dementia (Armstrong et al., 2019), so it is unclear why persons living with dementia with swallowing symptoms were less likely to access hospice. It is possible that choking associated with swallowing difficulties can lead to decisional conflict between continuing oral feeding and initiating tube feedings, potentially delaying, or deterring admission to hospice care programs (Dimech et al., 2020; Luth et al., 2021). Persons living with dementia are also more likely to experience hospital transitions compared to those without dementia (Shepherd et al., 2019), which may also deter hospice transitions. Because machine learning approaches sometimes reveal unanticipated relationships among data features such as the role of swallowing in hospice use, further investigation into the relationship between swallowing difficulty among persons living with dementia and hospice use is warranted.

Nonreliance on food stamps and having a regular physician predict hospice, which suggests that the privilege of receiving hospice care is not necessarily afforded to all (Corpora, 2021). There are significant income differences by age, household type, nativity, geographic region, and race/ethnicity, which can contribute to SDH, including access to health care resources. Householders less than 65 years old enjoy a median income 40 percent higher than those 65 and older (Semega et al., 2020, September 15). Thus, the cost of hiring support for in-home care is insurmountable for the majority of people, particularly among older, poor, and medically underserved families (Ornstein et al., 2021; Ornstein et al., 2017; Ornstein et al., 2019). Moreover, reliance on food stamps may be an indication of financial need that can serve as a proxy for Medicaid eligibility. This has important implications for the role of Medicaid in accessing hospice care given the disincentive for nursing homes to refer Medicaid recipients to hospice based on current reimbursement policies (Stearns et al., 2018).

Having sufficient financial resources, regular access to medical care, and an established, trusting relationship with a health care provider may help facilitate the acceptance of hospice enrollment (Sullivan et al., 2021). Furthermore, poor communication may perpetuate health inequity by reducing opportunities to understand the wants and needs of persons living with dementia and their families (Hart et al., 2018). A recent study of dementia caregivers who felt unprepared and unsure of what to expect, for example, reported that they typically were the ones to initiate conversations with their physicians about end-of-life care options (Obermeyer et al., 2015). Providing physicians and nurses with palliative care education can increase the use of hospice care, which suggests that integrating educational approaches in learning health systems may help to reduce barriers to care (Cardona et al., 2022; Chuang et al., 2021).

Whether health prevents persons living with dementia from enjoying life was also associated with hospice use. Health-related quality-of-life is a known predictor of mortality risk; particularly, among persons receiving dialysis due to kidney failure, which is a known complication of diabetes (Kalantar et al., 2019). Moreover, having multiple comorbid chronic health conditions has been associated with poorer quality-of-life among persons living with dementia, which is common with advanced age and illness, particularly among disadvantaged persons (Nelis et al., 2018).

Interestingly, persons living with dementia in this study with a good memory were better able to access hospice care. This is an important finding because nearly half of all hospice patients have a dementia diagnosis (Aldridge et al., 2021); yet there are very few studies investigating the extent to which persons living with dementia are able to articulate their wishes for end-of-life care (Song et al., 2019), how to best engage persons living with dementia in planning and preparing for end-of-life care (Bryant et al., 2019, Geshell et al., 2019), or how to best communicate end-of-life care preferences between persons living with dementia and their caregivers (Givens et al., 2018). Moreover, there is a lack of consensus on when to initiate specialist palliative care services despite the progressive functional and cognitive decline associated with dementia, which often leads to significant symptom burdens on the persons living with dementia and as the condition progresses (Mo et al., 2021).

### RF Final Model

RF was the most parsimonious model; however, it had lower predictive values than IGR and did not provide much useful clinical information (Supplementary Table 2). Despite this, findings produced by RF are certainly worth mentioning. As would be expected, increasing age of both persons living with dementia and their caregiver was associated with hospice. Persons living with dementia income and census division also predicted hospice, which is consistent with the IGR model and other research indicating that where a person lives contributes to end-of-life disparities (Rush et al., 2017). For example, people who live in socioeconomically disadvantaged neighborhoods are more likely to be discharged from hospice if they are admitted to the hospital while receiving hospice care compared to residents of other neighborhoods (Russell et al., 2020). This is consistent with other research revealing inverse relationships between poverty and hospice access (Riggs et al., 2016). Beyond neighborhoods, there are state-level variations in the availability of hospices, physicians, hospital beds, and home health care (Wang et al., 2015). The number of days of help per month the caregiver provides in the RF model is consistent with prior research indicating that dementia caregivers provide more hours of care over longer periods of time, without any additional support, particularly during end-of-life caregiving (Ornstein et al., 2019; Vick et al., 2019). Thus, it is clear that policy change is urgently needed to break down barriers to supportive care options for persons living with dementia and their family caregivers (Ornstein et al., 2021).

This study has important limitations. First, because hospice care is delivered differently based on the setting (e.g., home, long-term care, or assisted living), there are barriers to care that are unique to each setting, such as caregiver availability, financial concerns, or the presence of hospital-based hospice care in certain communities that we did not investigate in the present study. This is an important limitation to consider in future research to differentiate predictors of hospice use by setting. Second, socioeconomic factors such as the association between food stamps and decreased hospice use have particular relevance for hospice availability in residential care facilities. This is because there is generally a disincentive for nursing homes to enroll their residents in hospice programs as current reimbursement models dictate that nursing home residents cannot receive rehabilitative care and hospice services simultaneously (Stearns et al., 2018). Moreover, if a resident elects hospice care, the cost of room and board defaults to either self-pay or Medicaid (Stearns et al., 2018). Although our study found a significant association between Medicaid and hospice use overall, we were not able to detect significant associations between Medicaid and hospice use by residential status (i.e., community or residential care). It is possible this is due to the fact that NHATS does not easily differentiate which person living with dementia is a long-term nursing home resident, and the sample size further limits the analysis. Certainly, additional research is necessary to explore this important topic.

Most importantly, any study predicting hospice use has a certain amount of unavoidable bias because minoritized individuals are historically under-represented in hospice. While we recognize this as a limitation, particularly when using secondary data to build predictive models for the management of population health (Obermeyer et al., 2019), this study provides a starting point for future research investigating important quality-of-life and SDH features of persons living with dementia and their caregivers. Our use of a prospective, nationally representative data set, supported by the NIA, helps reduce bias. NHATS uses a stratified sampling approach, oversamples older adults, African Americans, and the prospective design limits recall bias. This study is also limited by the relatively few SDH factors available within the NHATS/NSOC, despite its comprehensiveness. Also, there are relatively few datapoints representing persons living with dementia and their primary caregivers available for investigating hospice, which underscores the challenges of conducting rigorous longitudinal research in this important area. Future research is urgently needed to determine how to best identify persons living with dementia in need of hospice care among vulnerable populations that may not be represented in the present study.

There are also limitations inherent in our methodological approach. Pearson’s correlation matrix has a number of limitations and assumptions, including the independence of association assumption, and the variables should be at the continuous level of measurement. Becasue this study involves both categorical and continuous features, the violation of this assumption may reduce accuracy. However, it is undeniable that this method provides important insights about the data set. Also, PCA is usually used for continuous and linear data. Although our data set does not fully satisfy this condition, it still reveals valuable insights. For example, rounds 5 and 7 use the same 61 features, but there are significant differences between them. This outcome further illustrates the need for multiyear analysis.

This study is novel because it explores an important, understudied area and provides new and useful insights into important features predicting hospice in dementia caregiving dyads over time. This study produced four predictive models, all providing a different lens through which to consider the contribution of quality-of-life and SDH features associated with hospice. Although we selected the IGR model based on its performance and clinical relevance, this study poses new and interesting considerations of potential drivers of hospice care, and the consistency of certain features using the different methods reveals some important commonalities for future research (e.g., caregiver, life enjoyment, and socioeconomic features). Because this study is exploratory, further research is needed before robust, actionable models can be translated into practice. Additional research is also necessary to investigate persons living with dementia who qualify for hospice care but do not transition to hospice for end-of-life care to further evaluate barriers to care. When translated into practice, data-driven models help achieve the vision of precision health in learning health systems by reducing structural barriers to hospice care transitions. The conclusions of this study may be used to understand an array of critical quality-of-life and SDH factors that may influence hospice care transitions for persons living with dementia, and aids in the development of new directions for research and practice concerning hospice care transitions.

## Supplementary Material

igac051_suppl_Supplementary_Material
