# Supplementary material for: Predicting Hospice Transitions in Dementia Caregiving Dyads: An Exploratory Machine Learning Approach
Source: Innov Aging. 2022 Aug 11;6(6):igac051. doi: 10.1093/geroni/igac051 (PMC9701063; doi:10.1093/geroni/igac051)
Supplement: igac051_suppl_Supplementary_Material [file igac051_suppl_supplementary_material.docx]

Supplementary Table 1. *Pearson’s Correlation Coefficients for Hospice in Round 5 and Round 7*

| *Round 5 (2015)* | |  | *Round 7 (2017)* | |
| --- | --- | --- | --- | --- |
|  | *pd8hospcelml* |  |  | *pd8hospcelml* |
| *pd8hospcelml* | *1* |  | *pd8hospcelml* | *1* |
| *hc5hartsrgyr* | *0.014564* |  | *hc7hartsrgyr* | *0.026357* |
| *hc5disescn7* | *0.081052* |  | *hc7disescn7* | *-0.09001* |
| *hc5disescn2* | *0.055528* |  | *hc7disescn2* | *-0.01385* |
| *hc5disescn6* | *-0.16221* |  | *hc7disescn6* | *-0.22248* |
| *hc5disescn8* | *-0.09622* |  | *hc7disescn8* | *0.075639* |
| *hc5disescn10* | *-0.00155* |  | *hc7disescn10* | *0.043026* |
| *hc5sleepmed* | *-0.1842* |  | *hc7sleepmed* | *0.010445* |
| *ss5probspeak* | *-0.00351* |  | *ss7probspeak* | *0.020364* |
| *ss5probchswl* | *0.016768* |  | *ss7probchswl* | *0.179976* |
| *sc5eathlp* | *-0.06266* |  | *sc7eathlp* | *-0.10234* |
| *hc5health* | *-0.19027* |  | *hc7health* | *-0.06554* |
| *ss5painlimts* | *-0.02525* |  | *ss7painlimts* | *-0.23253* |
| *ss5prbbrlimt* | *-0.11187* |  | *ss7prbbrlimt* | *-0.03509* |
| *cca5hwofthom* | *0.113235* |  | *cca7hwofthom* | *0.091095* |
| *hc5aslep30mn* | *-0.19019* |  | *hc7aslep30mn* | *-0.09479* |
| *hw5lst10pnds* | *-0.12202* |  | *hw7lst10pnds* | *0.063453* |
| *hc5depresan1* | *0.052241* |  | *hc7depresan1* | *-0.01059* |
| *hc5depresan2* | *-0.04178* |  | *hc7depresan2* | *-0.14626* |
| *hc5depresan3* | *-0.0534* |  | *hc7depresan3* | *-0.16581* |
| *hc5depresan4* | *0.043968* |  | *hc7depresan4* | *-0.20062* |
| *mc5medsmis* | *-0.15452* |  | *mc7medsmis* | *-0.0044* |
| *cg5ratememry* | *0.045961* |  | *cg7ratememry* | *-0.29959* |
| *pa5hlkpgoenj* | *0.150813* |  | *pa7hlkpgoenj* | *-0.24841* |
| *wb5offelche3* | *-0.09523* |  | *wb7offelche3* | *-0.34144* |
| *wb5truestme3* | *-0.02211* |  | *wb7truestme3* | *-0.18615* |
| *wb5agrwstmt1* | *0.033707* |  | *wb7agrwstmt1* | *-0.21223* |
| *hc5worrylimt* | *-0.01377* |  | *hc7worrylimt* | *-0.1314* |
| *mo5outoft* | *0.042075* |  | *mo7outoft* | *0.088409* |
| *fl5noonetalk* | *-0.14451* |  | *fl7noonetalk* | *-0.19914* |
| *sn5dnumsn* | *0.027065* |  | *sn7dnumsn* | *-0.23389* |
| *pa5htkfrrlsr* | *0.207986* |  | *pa7htkfrrlsr* | *-0.19767* |
| *pa5hlkepfvst* | *0.105957* |  | *pa7hlkepfvst* | *-0.08329* |
| *pa5hlkpfrvol* | *0.070014* |  | *pa7hlkpfrvol* | *0.005039* |
| *ew5progneed1* | *0.109851* |  | *ew7progneed1* | *0.061321* |
| *ew5finhlpfam* | *-0.15213* |  | *ew7finhlpfam* | *0.027116* |
| *mc5havregdoc* | *0.137201* |  | *mc7havregdoc* | *-0.0864* |
| *che5enrgylmt* | *-0.08297* |  | *che7enrgylmt* | *-0.0146* |
| *cac5diffphy* | *0.201469* |  | *cac7diffphy* | *-0.0581* |
| *cac5exhaustd* | *0.071369* |  | *cac7exhaustd* | *-0.01924* |
| *cac5diffinc* | *0.02231* |  | *cac7diffinc* | *-0.09147* |
| *cac5toomuch* | *0.110132* |  | *cac7toomuch* | *0.002231* |
| *cac5uroutchg* | *0.148813* |  | *cac7uroutchg* | *-0.05789* |
| *cac5notime* | *0.027222* |  | *cac7notime* | *-0.05114* |
| *cac5diffemlv* | *-0.20751* |  | *cac7diffemlv* | *-0.028* |
| *cpp5hlpkptgo* | *0.122705* |  | *cpp7hlpkptgo* | *-0.01525* |
| *che5health* | *-0.20786* |  | *che7health* | *-0.00531* |
| *che5sleepint* | *0.030801* |  | *che7sleepint* | *-0.07609* |
| *op5numhrsday* | *-0.03288* |  | *op7numhrsday* | *0.112901* |
| *op5numdaysmn* | *-0.12361* |  | *op7numdaysmn* | *0.062599* |
| *op5leveledu* | *0.001445* |  | *op7leveledu* | *0.016703* |
| *op5age* | *-0.02539* |  | *op7age* | *0.090438* |
| *r5d2intvrage* | *0.293289* |  | *r7d2intvrage* | *0.220968* |
| *op5relatnshp* | *0.06811* |  | *op7relatnshp* | *0.029791* |
| *r5dgender* | *0.182794* |  | *r5dgender* | *-0.01738* |
| *hh5martlstat* | *0.063039* |  | *hh7martlstat* | *0.036101* |
| *re5dcensdiv* | *0.049544* |  | *re7dcensdiv* | *0.018514* |
| *hh5dhshldchd* | *-0.10075* |  | *hh7dhshldchd* | *-0.00461* |
| *ia5totinc* | *-0.16257* |  | *ia7totinc* | *0.147672* |
| *hc5hosptstay* | *0.136491* |  | *hc7hosptstay* | *-0.06104* |
| *hc5hosovrnht* | *-0.13408* |  | *hc7hosovrnht* | *0.009753* |
| *SPrace* | *0.054293* |  | *SPrace* | *-0.23938* |

| Supplementary Table 2. *Model Performance Comparison* | | | | | |
| --- | --- | --- | --- | --- | --- |
| Model Performance | Single Round 5 | Single Round 7 | Rounds  5 and 7 | Information Gain Ratio Important Features  Rounds 5 and 7 | Random Forest Feature Importance Calculation  Rounds 5 and 7 |
| Average Accuracy | 0.598 | 0.624 | 0.597 | 0.685 | 0.624 |
| Average Sensitivity | 0.797 | 0.789 | 0.775 | 0.824 | 0.713 |
| Average Specificity | 0.386 | 0.416 | 0.401 | 0.537 | 0.557 |
| Average AUC | 0.595 | 0.645 | 0.602 | 0.743 | 0.703 |

*Note.* AUC=Area Under the Curve


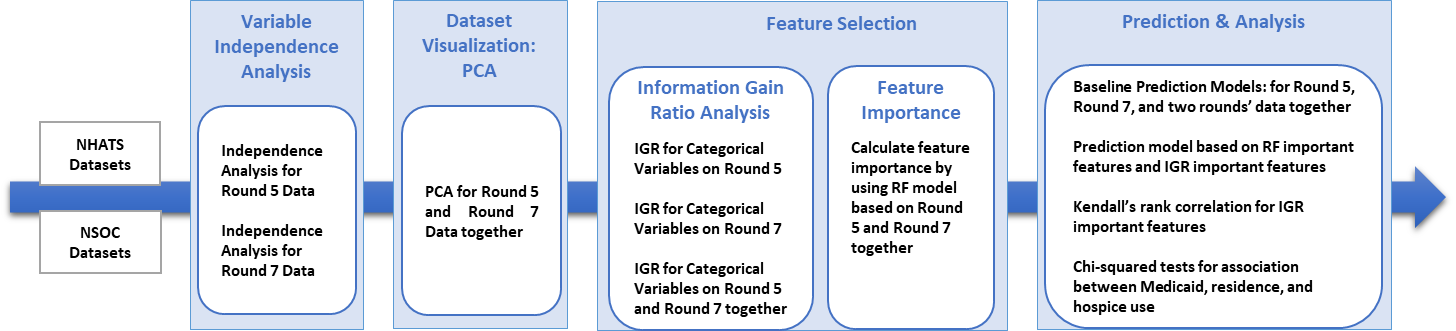


Supplementary Figure 1. *Study flowchart and Logic*


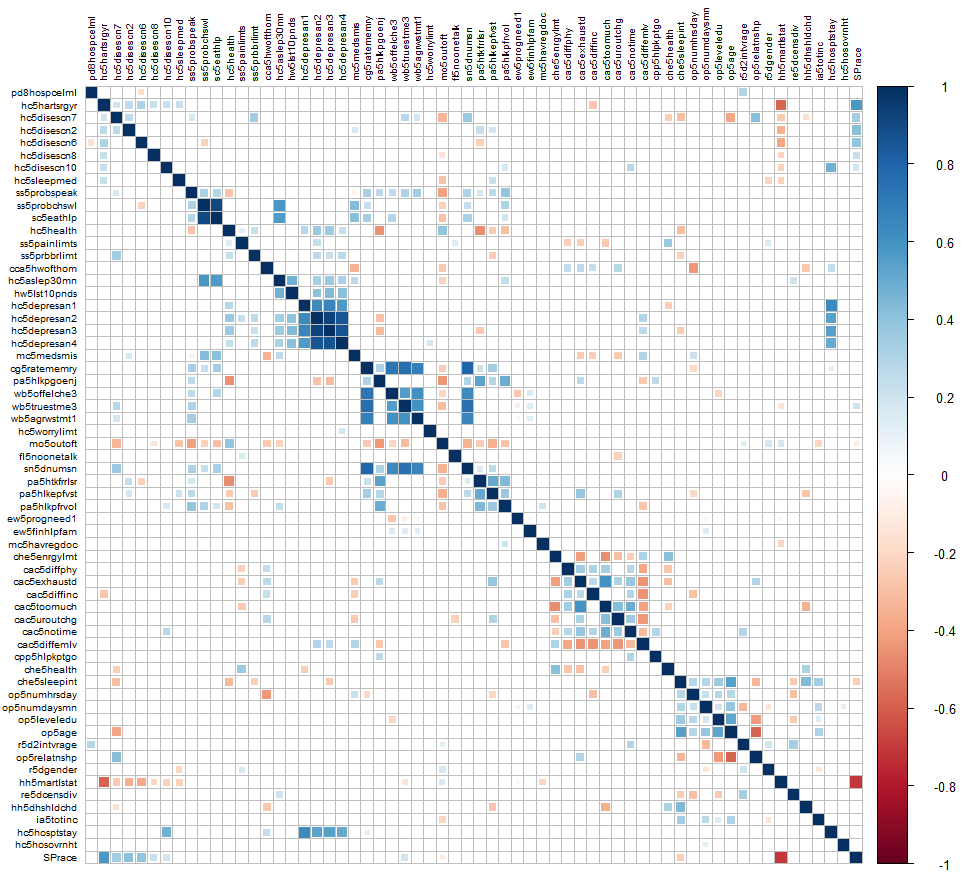


Supplementary Figure 2 (a). *Pearson’s Correlation Heatmaps for Dimensionality Reduction in Round 5 (2015)*

**Round 5** **hospice associations:** Persons living with dementia older age and diabetes.


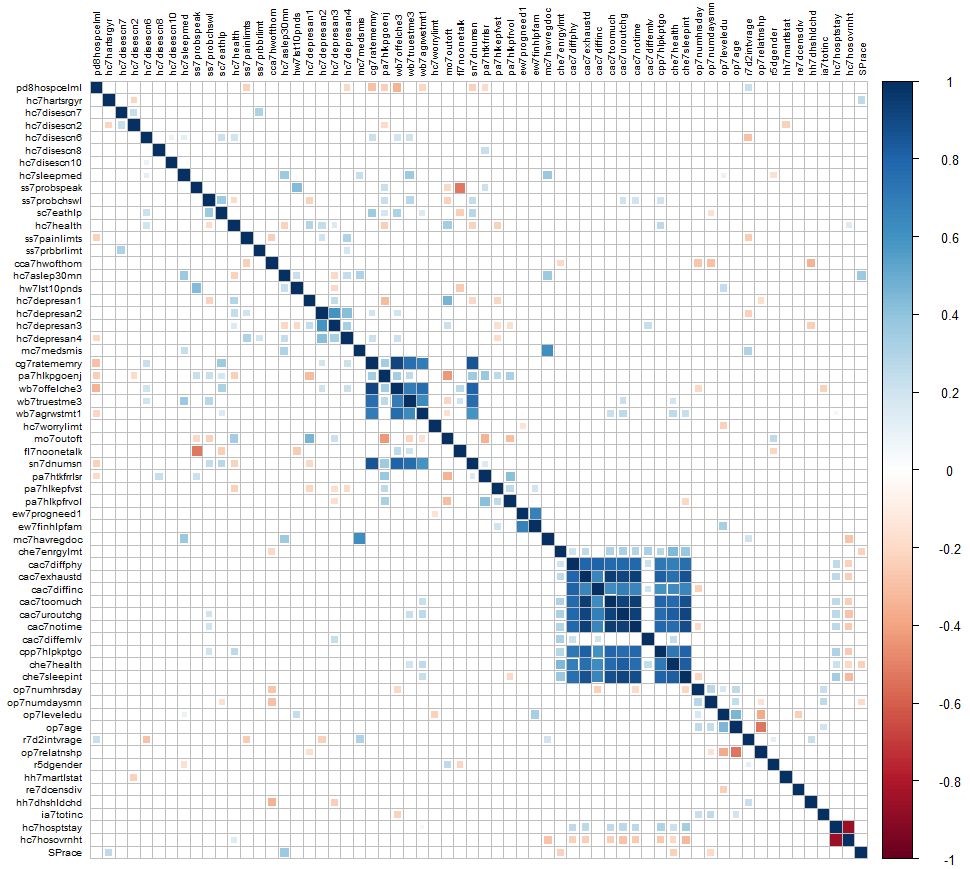


Supplementary Figure 2 (b). *Pearson’s Correlation Heatmaps for Dimensionality Reduction in Round 7 (2017)*

**Round 7 hospice associations:** Persons living with dementia older age; worrying less, feeling full of life, pain limits activity, health prevents enjoying life, health keeps from attending religious services, other people determine most of what can and cannot do, having fewer people in social network, and a good memory.

| **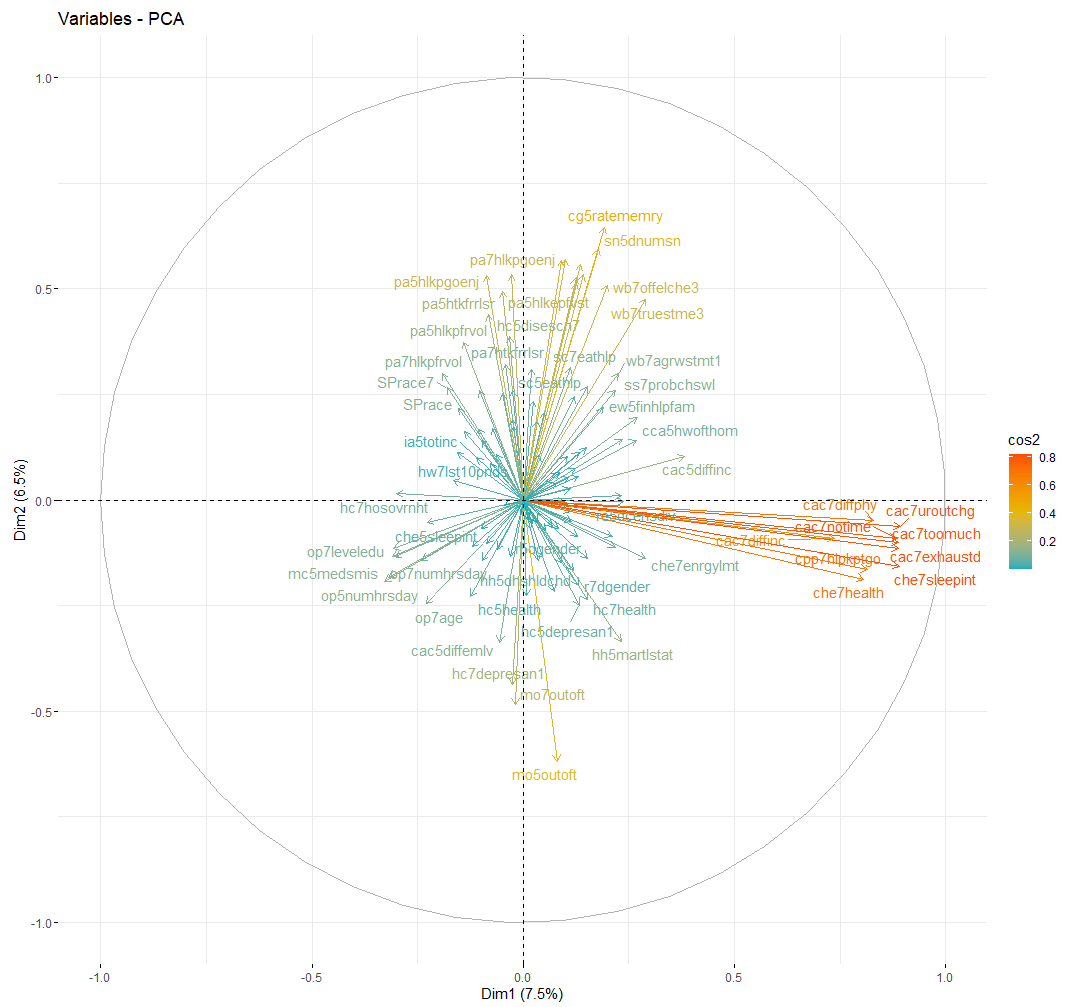***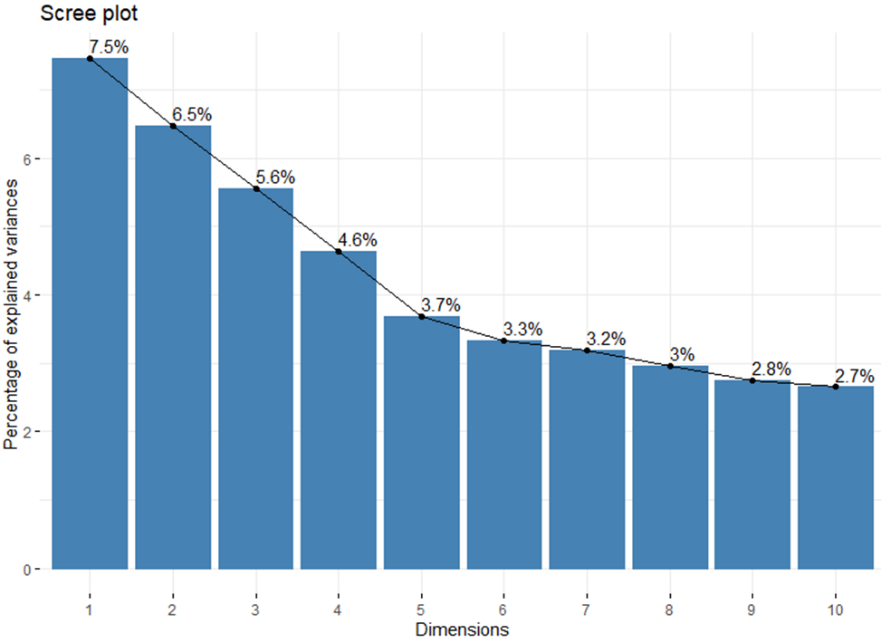* |
| --- |

Supplementary Figure 3. *Principal Component Analysis with Scree Plot*

| 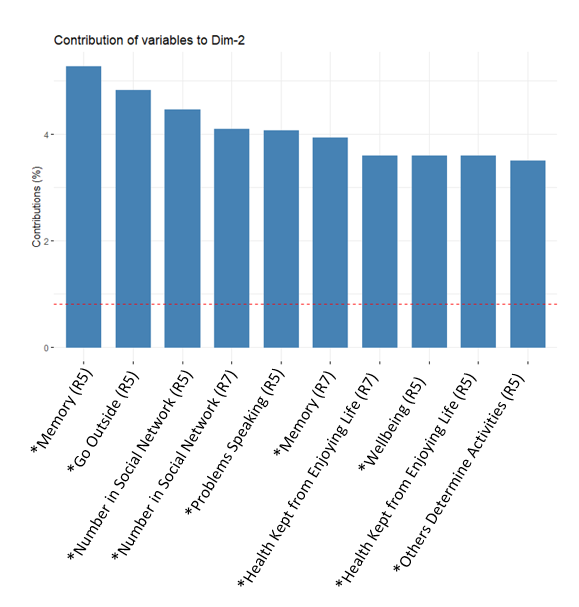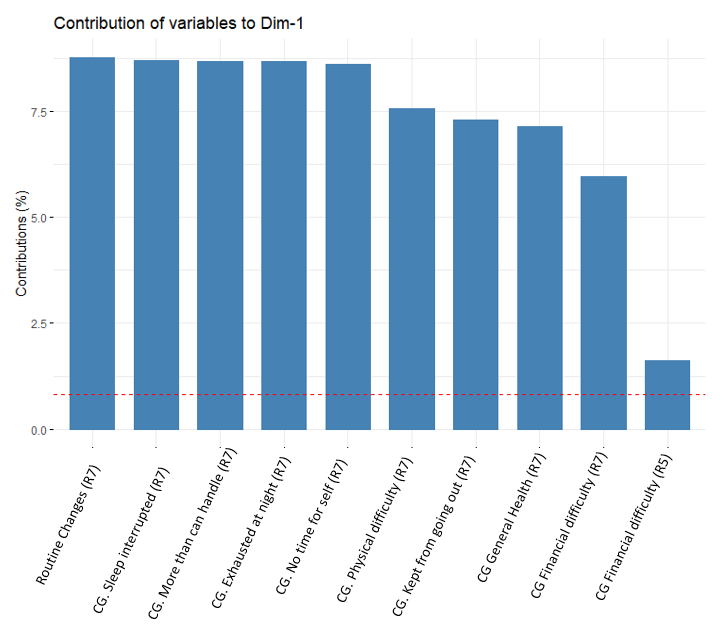 |
| --- |

Supplementary Figure 4. *Contribution of Important Features*

*Note.* *Indicates Person Living with Dementia. CG=caregiver


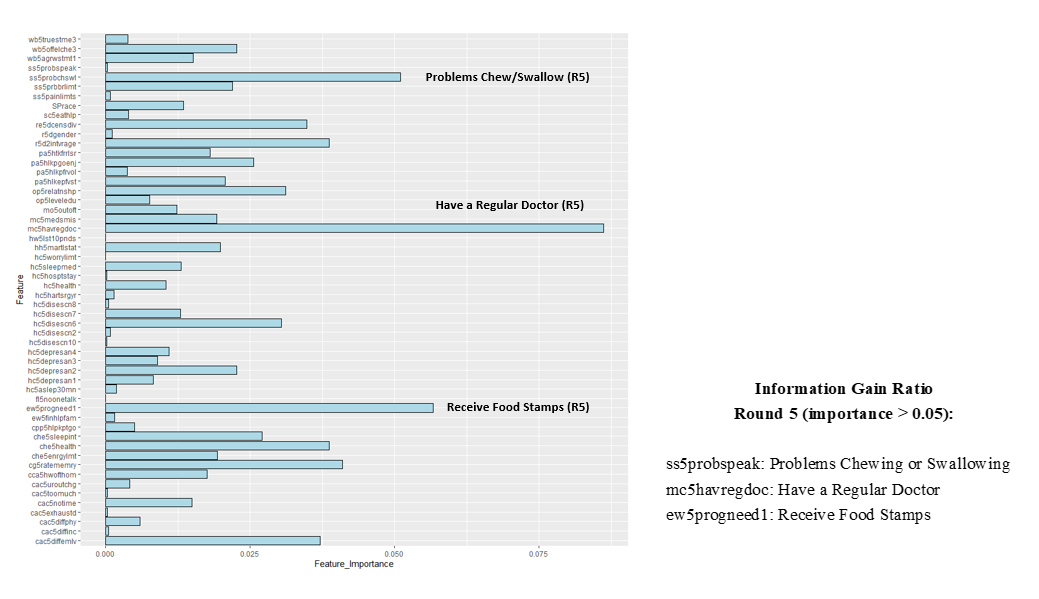


Supplementary Figure 5 (a). *Information Gain Ratio of Important Features in Round 5*


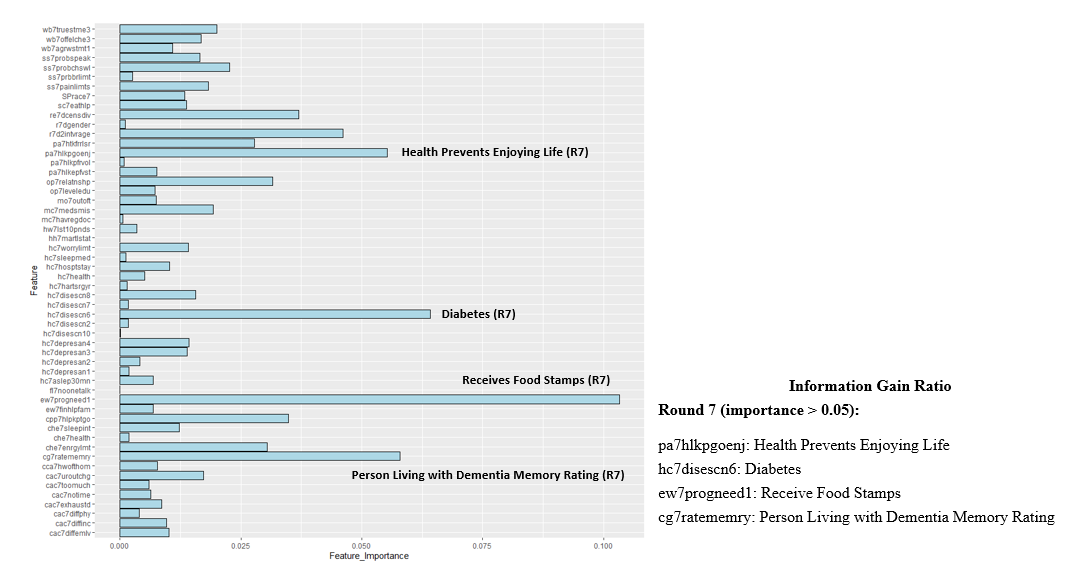


Supplementary Figure 5 (b). *Information Gain Ratio of Important Features in Round 7*


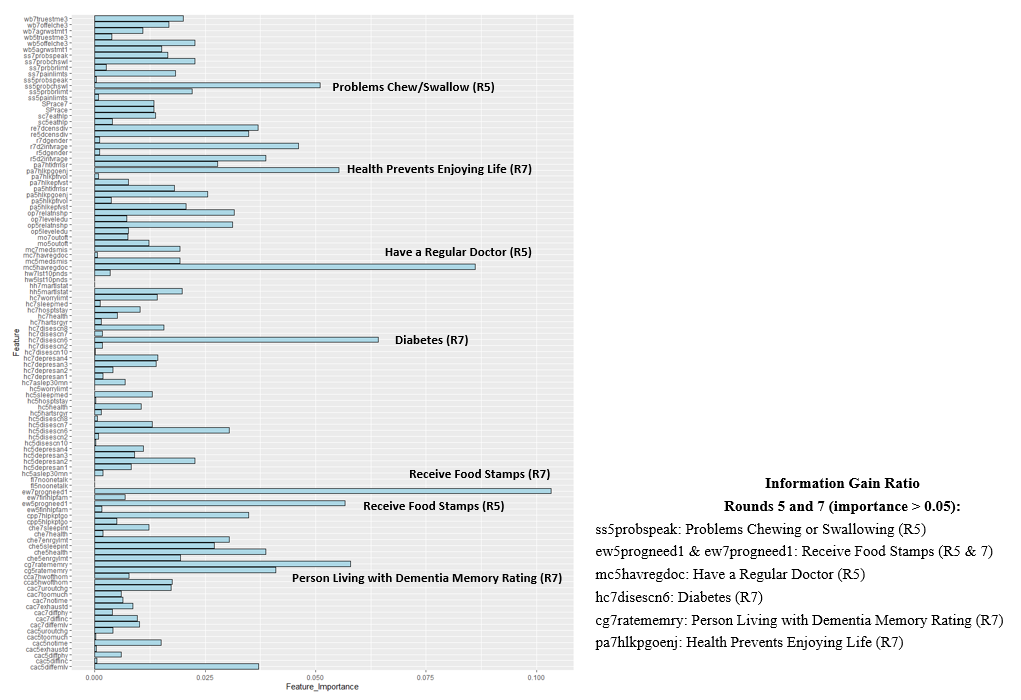


Supplementary Figure 5 (c). *Information Gain Ratio of Important Features in rounds 5 and 7*

| 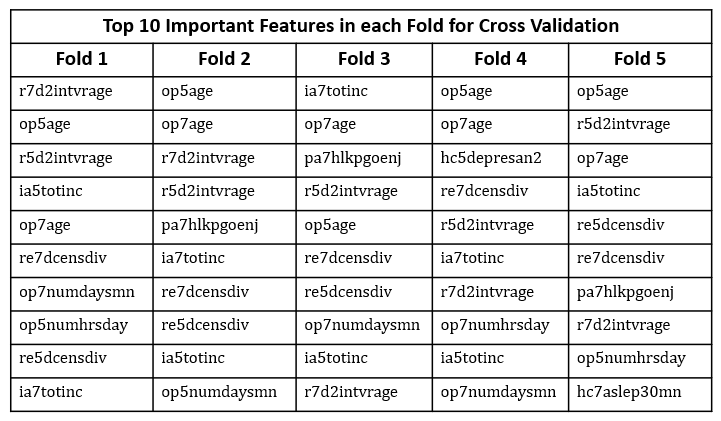 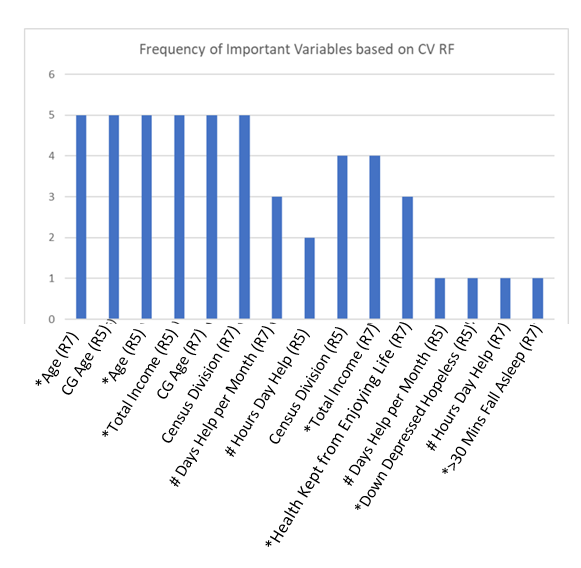 |
| --- |

Supplementary Figure 6. *Random Forest* *Feature Importance for Round 5 and Round 7*

Caregiver age (R5 & R7), total income (R7); Persons living with dementia age (R5 & R7); census division (R7); number of days per month caregiving (R7); health keeps from enjoying life (R7)

*Note.* *Indicates Person Living with Dementia. CG=caregiver
